# SGLT-2 inhibitors in cancer patients with diabetes: when cardioprotection is key

**DOI:** 10.1093/ehjcvp/pvaf038

**Published:** 2025-05-21

**Authors:** Mattia Brambilla, Bianca Larroux, Aldo Bonaventura

**Affiliations:** Internal Medicine Residency Program, School of Medicine, University of Insubria, Via Ottorino Rossi 9, 21100 Varese, Italy; Internal Medicine Residency Program, School of Medicine, University of Insubria, Via Ottorino Rossi 9, 21100 Varese, Italy; Medical Center, S.C. Medicina Generale 1, Department of Internal Medicine, Ospedale di Circolo and Fondazione Macchi, ASST Sette Laghi, Viale Borri 57, 21100 Varese, Italy

**This editorial refers to ‘Effects of sodium–glucose cotransporter 2 inhibitors in patients with cancer and diabetes mellitus: a systematic review and meta-analysis’, by G. Novo *et al*., https://doi.org/10.1093/ehjcvp/pvaf028.**

Cancer and cardiovascular (CV) diseases represent a conundrum that largely contributes to morbidity and mortality worldwide.^1^ While on active treatment, oncologic patients may develop cancer therapy-related cardiac dysfunction (CTRCD), whose main clinical manifestation is represented by heart failure (HF).^2^

In past years, a number of randomized controlled trials (RCTs) demonstrated the efficacy of sodium–glucose cotransporter-2 (SGLT-2) inhibitors in reducing major adverse CV events as well as HF hospitalizations in patients with type 2 diabetes at high CV risk. Importantly, these benefits were then extended also to patients without type 2 diabetes.^3^ Recently, the beneficial effects of SGLT-2 inhibitors were observed in patients with transthyretin cardiac amyloidosis.^4^ Whether the cardioprotective effects of SGLT-2 inhibitors apply also to the cardio-oncology field has been and still is a matter of research. Although no RCTs were conducted in cardio-oncology patients, a number of observational studies provided encouraging results in terms of lower risk of acute HF exacerbations, reduced all-cause mortality and all-cause hospitalizations/emergency department visits.^5,6^

Building on this growing evidence, the current issue of the *European Heart Journal—Cardiovascular Pharmacotherapy* reported the findings of the systematic review and meta-analysis by Novo *et al.* about the effects of SGLT-2 inhibitors in patients with cancer and type 2 diabetes.^7^ By including 11 observational studies with a total of 104 327 patients, the use of SGLT-2 inhibitors was associated with a 53% reduction in all-cause mortality and a 56% reduction in HF hospitalizations compared with patients who did not receive SGLT-2 inhibitors (*Figure 1*). These findings remained consistent with regard to all-cause mortality in patients receiving anthracyclines and in those without HF at baseline despite high heterogeneity among the studies, while the benefit in reducing hospitalizations for HF was confirmed in patients receiving anthracyclines with low heterogeneity, but not in patients without HF at baseline.^7^

**Figure 1:**
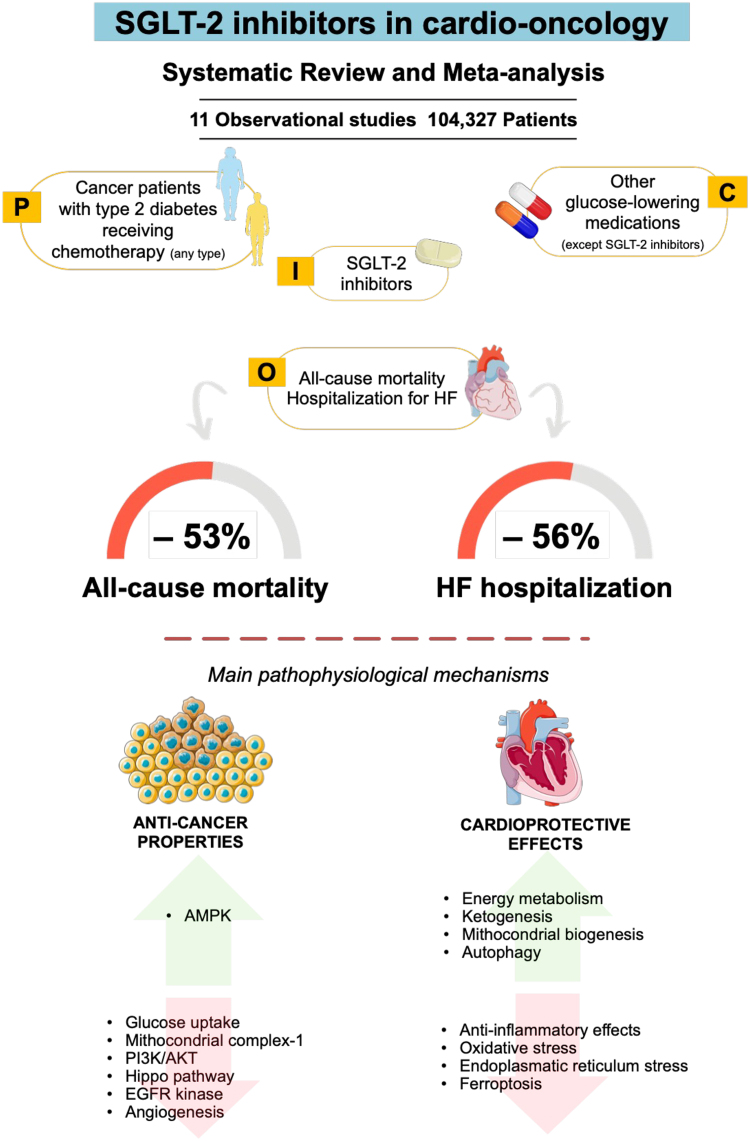
SGLT-2 inhibitors in cardio-oncology. SGLT-2 inhibitors provided beneficial results in terms of HF hospitalizations and all-cause mortality in patients with cancer and type 2 diabetes receiving chemotherapy. A number of pathophysiological mechanisms may explain anticancer properties and cardioprotective effects, including a reduction in fatty acid and protein synthesis and angiogenesis as well as enhanced cardiac metabolism and autophagy and reduced oxidative stress and inflammation. An exhaustive overview on these mechanisms can be found elsewhere.^8,9^ AKT, protein kinase B; AMPK, adenosine monophosphate-activated protein kinase; EGFR, epidermal growth factor receptor; HF, heart failure; PI3K, phosphoinositide 3-kinase; SGLT-2, sodium–glucose cotransporter-2. This figure has been created using Servier Medical Art templates, which are licensed under a Creative Commons Attribution 3.0 Unported License; https://smart.servier.com.

The results of the meta-analysis by Novo *et al.* are particularly interesting, as they support a therapeutic strategy in a field that currently lacks established pharmacological options. Compared with two previous meta-analyses,^10,11^ Novo *et al.* included a larger number of patients and a sub-analysis looking at patients without HF at baseline in addition to the one for patients treated with anthracyclines. They did not find any differences in terms of adverse events,^7^ confirming prior evidence.^10,11^ Therefore, the observational nature of the studies herein included may pre-dispose to bias, thus claiming for dedicated RCTs. To date, only a single RCT is available in this setting—the EMPACARD-PILOT trial (Use of EMPAgliflozin in the prevention of CARDiotoxicity)—that enrolled 76 patients with breast cancer receiving anthracycline-based chemotherapy without HF, where empagliflozin showed a reduction in CTRCD after 6 months of treatment.^12^

Despite the positive results provided by the meta-analysis by Novo *et al.*,^7^ their interpretation should be approached with caution, as correctly acknowledged by the authors. Indeed, a major concern is represented by the considerable heterogeneity among the studies in terms of patient characteristics, cancer type, and treatment regimens. In addition, all studies included in the meta-analysis by Novo *et al.* reported all-cause mortality. In cancer patients, it is relevant to discriminate between death due to a CV event or cancer progression since these two entities are closely intertwined and could detrimentally influence with each other.^1^ Accordingly, the issue of competing risks may explain the lack of significant reduction in HF hospitalizations among patients without baseline HF together with shorter follow-up periods (ranging from 1 to 4.8 years). Last but not least, guideline-directed medical therapies for HF were generally prescribed in only 30% to 50% of patients in the studies considered for the meta-analysis. However, renin–angiotensin–aldosterone blockers are supported by robust evidence in this population^13^ and this may have influenced the outcomes.

SGLT-2 inhibitors represent a therapeutic advancement of utmost importance in the treatment of patients with HF, irrespective of left ventricular ejection fraction, diabetes, and chronic kidney disease. These findings and the use of such treatments in patients with amyloidosis^4^ further support their beneficial effects across a broad spectrum of HF aetiologies. Indeed, other than diuresis and natriuresis, SGLT-2 inhibitors provide cardioprotective, anticancer, and anti-inflammatory effects^8^ that explain the improvement in outcomes, such as acute HF, all-cause mortality, all-cause hospitalizations, atrial fibrillation/flutter, and acute kidney injury.^14^

In conclusion, the meta-analysis by Novo *et al.* offers promising evidence about SGLT-2 inhibitors in the cardio-oncology field. Dedicated RCTs are eagerly needed to further confirm the encouraging findings of observational studies, irrespective of diabetes status. To this end, some RCTs are currently ongoing to test SGLT-2 inhibitors in patients receiving anthracycline-based therapies (dapagliflozin: NCT06341842, NCT06427226, NCT06304857; empagliflozin: NCT05271162, NCT06103279). Additional research might also explore in more detail underlying molecular mechanisms of SGLT-2 inhibitors in order to help stratify and phenotype patients with cancer receiving cardiotoxic therapies. Beside this, in light of the beneficial role of glucagon-like peptide-1 receptor agonists (GLP-1 RAs) in patients with HF, accumulating evidence suggests cardioprotective effects. In patients with obesity or type 2 diabetes undergoing chemotherapy with anthracyclines, lower incidence of HF exacerbation, hospitalizations, and mortality was reported.^15^ In case these encouraging findings are confirmed, the possible association of SGLT-2 inhibitors and GLP-1RAs in oncologic patients with CTRCD could result in a net improvement of CV outcomes.

## Data Availability

The present manuscript does not contain any original data.
